# Genomic regions and signaling pathways associated with indicator traits for feed efficiency in juvenile Atlantic salmon (*Salmo salar*)

**DOI:** 10.1186/s12711-020-00587-x

**Published:** 2020-11-06

**Authors:** Hanne Dvergedal, Thomas Nelson Harvey, Yang Jin, Jørgen Ødegård, Lars Grønvold, Simen Rød Sandve, Dag Inge Våge, Thomas Moen, Gunnar Klemetsdal

**Affiliations:** 1 Department of Animal and Aquacultural Sciences, Faculty of Biosciences, Norwegian University of Life Sciences, P. O. 5003, 1433 Aas, Norway; 2 AquaGen AS, P. O. 1240, 7462 Trondheim, Norway

## Abstract

**Background:**

One objective of this study was to identify putative quantitative trait loci (QTL) that affect indicator phenotypes for growth, nitrogen, and carbon metabolism in muscle, liver, and adipose tissue, and for feed efficiency. Another objective was to perform an RNAseq analysis (184 fish from all families), to identify genes that are associated with carbon and nitrogen metabolism in the liver. The material consisted of a family experiment that was performed in freshwater and included 2281 individuals from 23 full-sib families. During the 12-day feed conversion test, families were randomly allocated to family tanks (50 fish per tank and 2 tanks per family) and fed a fishmeal-based diet labeled with the stable isotopes ^15^N and ^13^C at inclusion levels of 2 and 1%, respectively.

**Results:**

Using a linear mixed-model algorithm, a QTL for pre-smolt growth was identified on chromosome 9 and a QTL for carbon metabolism in the liver was identified on chromosome 12 that was closely related to feed conversion ratio on a tank level. For the indicators of feed efficiency traits that were derived from the stable isotope ratios (^15^N and ^13^C) of muscle tissue and growth, no convincing QTL was detected, which suggests that these traits are polygenic. The transcriptomic analysis showed that high carbon and nitrogen metabolism was associated with individuals that convert protein from the feed more efficiently, primarily due to higher expression of the proteasome, lipid, and carbon metabolic pathways in liver. In addition, we identified seven transcription factors that were associated with carbon and nitrogen metabolism and located in the identified QTL regions.

**Conclusions:**

Analyses revealed one QTL associated with pre-smolt growth and one QTL for carbon metabolism in the liver. Both of these traits are associated with feed efficiency. However, more accurate mapping of the putative QTL will require a more diverse family material. In this experiment, fish that have a high carbon and nitrogen metabolism in the liver converted protein from the feed more efficiently, potentially because of a higher expression of the proteasome, lipid, and carbon metabolic pathways in liver. Within the QTL regions, we detected seven transcription factors that were associated with carbon and nitrogen metabolism.

## Background

Sustainable aquaculture production depends on the efficient conversion of feed resources into high-quality products [1]. In Norway, the feed costs of salmonid production represented ~ 50% of the total production costs in 2017, totaling ~ 2.2 billion euros [2]. This means that an improvement in feed efficiency has considerable value. Feed efficiency can be defined as the ratio of output to input (feed efficiency ratio, FER), i.e. as the ratio of growth to the unit of feed consumed, whereas feed conversion ratio (FCR) is the inverse of this ratio. In fish, genetic variance exists for fecal losses, due to individual variation in digestibility [3]. Genetic variance exists also for maintenance (degradation and replacement of previously deposited protein), and for growth, if the composition of growth is ignored, i.e. for protein metabolism [4]. The relative contribution of nutrients to protein metabolism can be assessed by using feed that is enriched with certain isotopes (i.e., with altered ratios of ^13^C/^12^C and/or ^15^N/^14^N) and monitoring the subsequent rate of change in the isotope profile of different tissues [5–7]. Nitrogen and carbon isotopes are the most relevant when assessing feed efficiency because all organic compounds contain carbon, while nitrogen is common to all amino acids. Molecules that contain ^14^N versus ^15^N differ in mass, and the ratio of these isotopes can be detected with an element analysis isotope ratio mass spectrometry system [8]. The genetic components of nitrogen and carbon metabolism in salmon were elucidated in an earlier study by Dvergedal et al. [4] by measuring the rate of change in the isotope profiles in different tissues. The isotope profiles were used to calculate indicators of FER/FCR (isotope-based FER (IFER)/ FCR (IFCR)). In addition, they (*r*_*g*_) of tank-FCR with indicator traits based on nitrogen and carbon metabolism in muscle tissue measured by using stable isotopes (^15^N and ^13^C) (*r*_*g*_ ~ 1.0), and with carbon metabolism in liver (ALC) (*r*_*g*_ ~ 0.9). These results are in accordance with those reported by Hawkins et al. [9], who proposed that differences in protein metabolism between individuals are genotype-dependent. Efficient fish are characterized by high protein growth and reduced protein degradation in muscle at the same relative growth rates [10]. In addition, Dvergedal et al. [4] showed that growth, isotope-based indicator traits, and sampling day jointly explained 73% of the observed variance in tank-FCR records, compared to 53–63% by growth and sampling day alone [4]. Hence, including nitrogen and carbon metabolism traits for relevant tissues substantially improved the prediction of FCR. Establishing the genetic basis of individual differences in feed utilization may have major implications for selection in aquaculture breeding programs. Moreover, genetic improvement of feed efficiency, by selection for growth or by other means, will decrease production costs and the environmental footprint per unit produced [11, 12].

To date, no genome-wide association study (GWAS) has reported quantitative trait loci (QTL) related to FER/FCR in Atlantic salmon [13, 14]. However, in beef cattle [15, 16], chicken [17–19], pigs [20, 21], and some other fish species [22, 23], QTL have been detected primarily for feed conversion efficiency or residual feed intake. Because individual phenotypic records are difficult to obtain, it is not easy to assess FER/FCR in aquatic species. With indicator phenotypes for nitrogen and carbon metabolism, feed efficiency can now be evaluated at the individual level and used in GWAS [24] and for marker-assisted selection [24, 25].

This study is based on a large-scale family experiment in Atlantic salmon, where families were kept separately in replicate tanks, with individual recording of growth and isotope profiles after feeding on ^15^N and ^13^C-enriched feed. The objective was to identify QTL that affect relevant indicator phenotypes for FER/FCR: weight gain (WG), relative weight gain (RG), atom % ^13^C in muscle (AMC), atom % ^15^N in muscle (AMN), atom % ^13^C in liver (ALC), atom % ^15^N in liver (ALN), atom % ^13^C in adipose tissue (AAC), the indicator trait of FCR for AMC (IFCR_AMC), the indicator trait of FCR for AMN (IFCR_AMN), the indicator trait of FER for AMC (IFER_AMC), and the indicator trait of FER for AMN (IFER_AMN) [4]. Another objective was to use RNAseq analysis to identify genes the expression of which is associated with carbon and nitrogen metabolism in the liver.

## Methods

### Phenotypic data

Phenotypic data on Atlantic salmon were collected from a family experiment that was carried out at the fish laboratory of the Norwegian University of Life Sciences (NMBU) in Aas, Norway. Details on this experiment are in Dvergedal et al. [4]. Broodstock from AquaGen’s breeding population (22 males and 23 females) were used to generate 23 families. To ensure clearly contrasted family groups with respect to growth potential and feed efficiency, divergent parents were selected based on high and low estimated breeding values (EBV) for growth in seawater.

Prior to the start of feeding, multiple families were kept in separate compartments within the same tank, with five tanks required to house all the families. Based on parentage assignment, 100 family members were identified for each of the 23 families and reared together in a single tank from the start of feeding until the start of the feed conversion test. Prior to the 12-day test, families were allocated to tanks, 50 fish per tank and two tanks per family (except for nine tanks in which the number of fish varied from 42 to 54 due to mortality prior to the start of the experiment and a counting mistake), for a total of 2281 fish. Families were fed a fishmeal-based diet labeled with the stable isotopes ^15^N and ^13^C at inclusion levels of 2 and 1%, respectively, as described by Dvergedal et al. [4]. Feed conversion rate was recorded at the family level.

Phenotypic data were recorded individually for WG, RG, AMC, AMN, ALC, ALN, and AAC, as described by Dvergedal et al. [4], resulting in phenotypes for 2249 to 2280 fish per trait. Muscle, liver, and adipose samples from each individual were collected in cryotubes and snap-frozen in liquid nitrogen for stable isotope analysis. The sampling procedure and determination of atom % ^15^N and ^13^C in the samples are explained in detail in Dvergedal et al. [4]. The stable isotope analysis was carried out at the Institute for Energy Technology (Kjeller, Norway).

From the individual ($i$) phenotypes of AMC and AMN, individual isotope-based indicator traits for FCR and FER (IFCR and IFER, respectively), i.e. ${IFCR\_AMC}_{i}$, ${IFCR\_AMN}_{i}$, ${IFER\_AMC}_{i}$, and ${IFER\_AMN}_{i}$, were derived as follows (taking ^15^N as an example):

$${IFCR\_AMN}_{i}=\frac{{FW}_{i}*{APE}_{Ni} }{{FW}_{i}-{IW}_{i}} \,\,{\text {and}}\,\, {IFER\_AMN}_{i}=\frac{{FW}_{i}-{IW}_{i}}{{FW}_{i}* {APE}_{Ni}},$$

where ${IW}_{i}$ and ${FW}_{i}$ is the initial and final weight of fish $i$, ${APE}_{Ni}={(AMN}_{i}-IA\%)$, with $IA$% equal to 0.370% for ^15^N and 1.087% for ^13^C. After a diet switch, the atom percentage in excess (APE) of a stable isotope in muscle tissue is expected to be proportional to the fraction of newly synthesized nutrients in the muscle, and the product of APE and final weight is expected to be proportional to the mass of new nutrients in the body tissue. Because IFCR is expected to be proportional to the amount of newly deposited body nutrients per g increase in body weight, fish that exchange a larger fraction of the body mass per unit of growth will be less feed-efficient. Exchange of body tissue is traceable with stable-isotope profiling and is related to the feed intake of the individual, the denominator of the ratio is weight gain, and the ratio between these two variables equals IFCR or the inverse equals IFER.

### Genotypic data

When the fish reached 5 to 10 g, they were pit-tagged with a 2 $\times$ 12 mm unique glass tag (RFID Solutions, Hafrsfjord, Norway), and a fin-clip was collected from 2300 fish for DNA-extraction and genotyping. Fin clips (20 mg) were incubated in lysis buffer and treated with proteinase K (20 µg/ml) at 56 ℃ overnight. The following day, DNA was isolated from the lysate at Biobank AS (Hamar, Norway) using the Sbeadex livestock kit (LGC Genomics) according to the manufacturer’s protocol (Thermo Fisher Scientific). DNA concentration was measured using a Nanodrop 8000 (Thermo Fisher Scientific). All fish were genotyped using AquaGen’s custom Axiom®SNP (single-nucleotide polymorphism) genotyping array from Thermo Fisher Scientific (former Affymetrix) (San Diego, CA, USA). This SNP-chip contains 56,177 SNPs that were originally identified based on Illumina HiSeq reads (10–15 × coverage) from 29 individuals from AquaGen’s breeding population. Genotyping was done at CIGENE (Aas, Norway). Genotypes were called from the raw data using the Axiom Power Tools software from Affymetrix. Individuals with a Dish-QC score lower than 0.82, and/or a call-rate lower than 0.97, and/or more than 10% missing genotypes were removed from further analyses. Also, SNPs with a minor allele frequency (MAF) lower than 1%, and with a missing call rate greater than 10% were removed. After filtering, 54,200 SNPs were included in the analysis.

### Association analysis

**Fig. 1:**
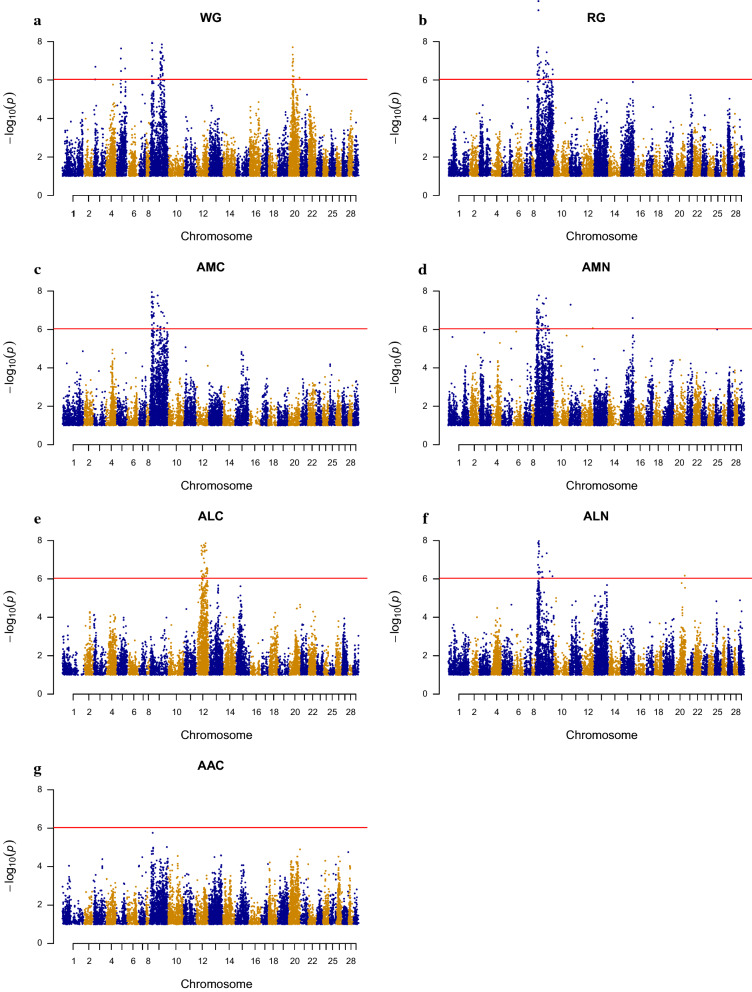
Genome-wide Manhattan plot for **a** weight gain (WG), **b** relative weight gain (RG), **c** atom % ^13^C muscle (AMC), **d** atom % ^15^N muscle (AMN), **e** atom % ^13^C liver (ALC), **f** atom % ^15^N liver (ALN), and **g** atom % ^13^C adipose tissue (AAC). The horizontal line represents the genome-wide Bonferroni −log10 (*p*) = 6.03 threshold

**Fig. 2:**
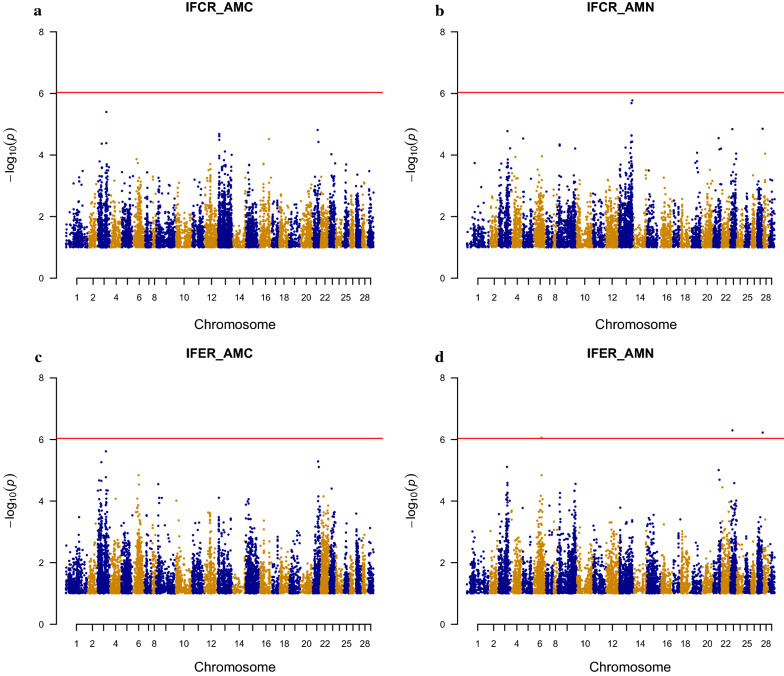
Genome-wide Manhattan plot for **a** indicator trait of feed conversion ratio for atom % ^13^C muscle (IFCR_AMC), **b** indicator trait of feed conversion ratio for atom % ^15^N muscle (IFCR_AMN), **c** indicator trait of feed efficiency ratio for atom % ^13^C muscle (IFER_AMC), and **d** indicator trait of feed efficiency ratio for atom % ^15^N muscle (IFER_AMN). The horizontal line represents the genome-wide Bonferroni −log10 (*p*) = 6.03 threshold

**Fig. 3:**
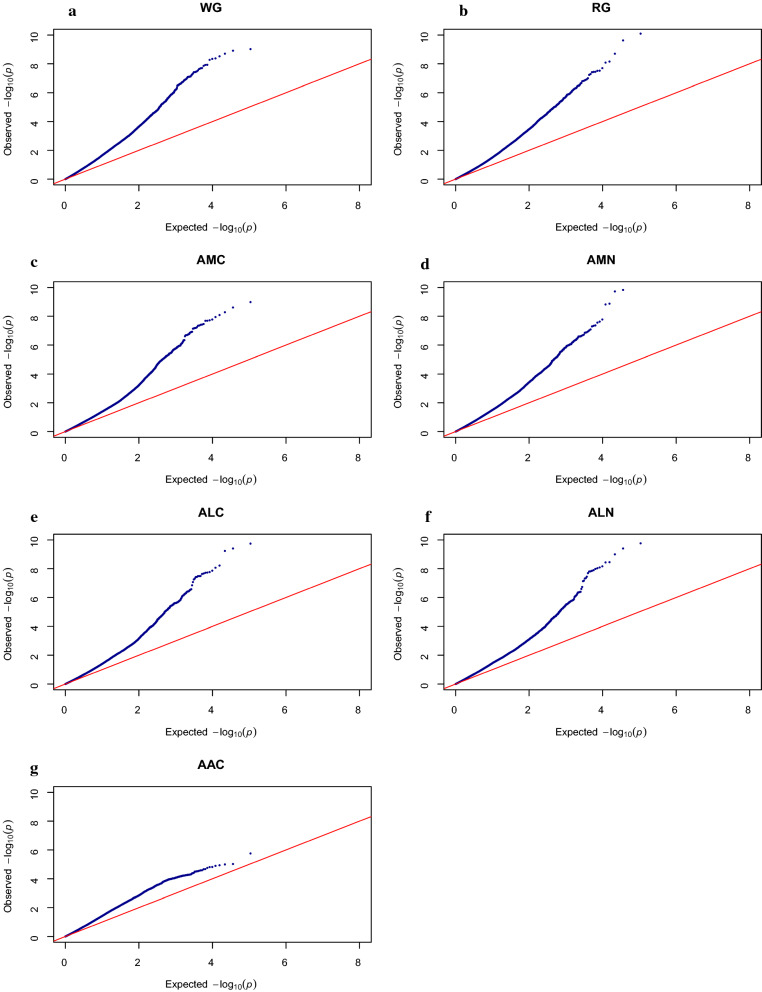
Q–Q plots for association analyses of **a** weight gain (WG), **b** relative weight gain (RG), **c** atom % ^13^C muscle (AMC), **d** atom % ^15^N muscle (AMN), **e** atom % ^13^C liver (ALC), **f** atom % ^15^N liver (ALN), and **g** atom % ^13^C adipose tissue (AAC)

**Fig. 4:**
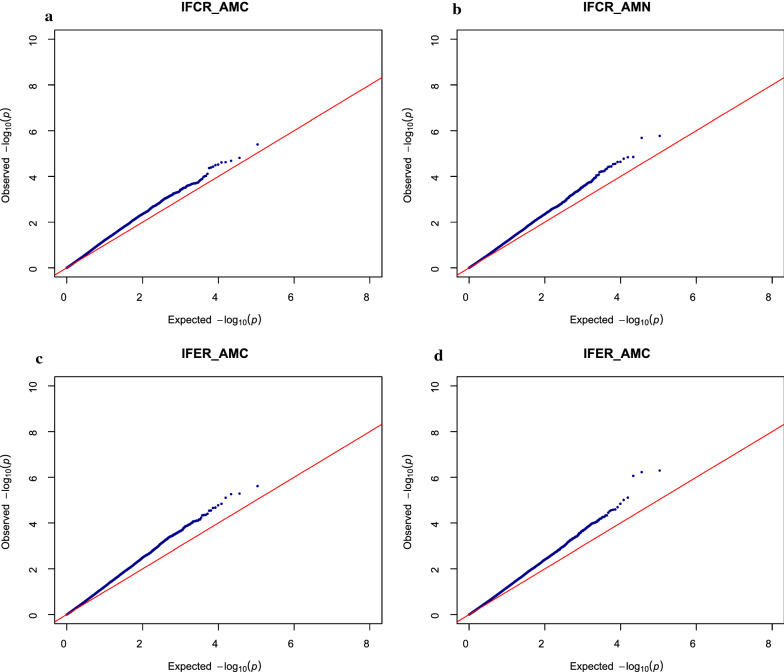
Q–Q plots for association analyses of the **a** indicator trait of feed conversion ratio for atom % ^13^C muscle (IFCR_AMC), **b** indicator trait of feed conversion ratio for atom % ^15^N muscle (IFCR_AMN), **c** indicator trait of feed efficiency ratio for atom % ^13^C muscle (IFER_AMC), and **d** indicator trait of feed efficiency ratio for atom % ^15^N muscle (IFER_AMN)

Associations between each SNP and the phenotypes related to nitrogen and carbon metabolism (AMC, AMN, ALC, ALN, and AAC), growth (WG, RG), and indicator traits of feed efficiency (IFCR_AMC, IFCR_AMN, IFER_AMC, and IFER_AMN) were tested by using a linear mixed-model algorithm implemented in the GCTA software [26]. The leave-one-chromosome-out option (–mlm-loco) was used, in which the chromosome that contains the tested SNP was left out when building the genetic relationship matrix (GRM). The linear mixed model used can be written:

$$Y_{i} = a + bx + g_{i}^{ - } + \varepsilon _{i} ,$$

where ${Y}_{i}$ is the phenotypes of individual $i$ for one of the evaluated traits, $a$ is the intercept, $b$ is the fixed regression for the SNP to be tested for association, $x$ is the SNP genotype indicator variable, coded as 0, 1 or 2, ${g}_{i}^{-}$ is the random polygenic effect for individual $i$, assumed to be distributed as ~ $N({\bf {0}}, \mathbf{G}{\sigma }_{g}^{2}$*)*, where $\mathbf{G}$ is the genomic relationship matrix, computed using the genotyped SNPs on all chromosomes except on the chromosome on which the tested SNP is located, ${\sigma }_{g}^{2}$ is the variance of the polygenic effect, and ${\varepsilon }_{i}$ is a random residual. In this algorithm, ${\sigma }_{g}^{2}$ is re-estimated each time a chromosome is left out from the calculation of the GRM. The level of significance was evaluated using a built-in likelihood-ratio test. The threshold value for 5% genome-wide significance was derived using the Bonferroni correction as 0.05/54,200 = 9.23 $\times$ 10^–7^, corresponding to a −log10 *p*-value (*p*) of 6.03. The number of SNPs on each chromosome was used to calculate 5% chromosome-wide significance levels. The Bonferroni correction is known to be overly conservative especially when applied to correlated SNP data, i.e., to SNPs that are in linkage disequilibrium, which can produce an excess of false-negative results [27]. Manhattan plots were used to visualize the −log10 (*p*) of SNPs across the chromosomes (n = 29) (Figs. 1 and 2) and QQ-plots were used to visualize the distribution of observed versus expected genome-wide −log10 (*p*) (Figs. 3 and 4).

### RNA extraction and sequencing

RNA was extracted from the liver of 184 fish, at ~ 10 months of age (four fish per tank), using the RNeasy Plus Universal Kit (Qiagen, Hilden, Germany) according to the manufacturer’s protocol. The RNA concentration was determined using a Nanodrop 8000 (Thermo Fisher Scientific, Waltham, USA), and RNA quality was determined using a 2100 Bioanalyzer with the RNA 6000 Nano Kit (Agilent Technologies, Santa Clara, USA). All samples had an RNA integrity number greater than 8. Sequencing libraries were constructed using the TruSeq Stranded mRNA Library Prep Kit (Illumina, San Diego, USA) according to the manufacturer’s instructions. Sequencing was performed on an Illumina Hiseq 2500 at the Norwegian Sequencing Center (Oslo, Norway), using 100 bp single-end sequencing. All raw fastq-files have been deposited on ArrayExpress under accession number E-MTAB-8305. Reads were trimmed, aligned to the salmon genome (ICSASG_v2), and counted using the bcbio-nextgen pipeline (https://github.com/bcbio/bcbio-nextgen) and the NCBI salmon genome annotation (https://salmobase.org/Downloads/Salmo_salar-annotation.gff3).

### RNAseq analysis

The EdgeR software [28] was used to identify genes that had an expression in the liver associated with the nitrogen and carbon metabolism liver traits (ALN and ALC), using the following linear regression model:

$$~Y_{{Fij}} = ~\mu _{F} + b_{1} x_{j} + b_{2} \left( {\frac{{\left( {FW_{i} ~ - ~IW_{i} } \right)}}{{FW_{i} }}} \right) + \varepsilon _{{Fij}} ,$$

where ${Y}_{Fij}$ is the expression of gene $i$ for fish $j$ in family $F$, ${\mu }_{F}$ is the intercept for each family, ${b}_{1}$ is the fixed regression coefficient of the phenotype for the trait (${x}_{j}$= ALC or ALN of fish $j$), and ${b}_{2}$ is the fixed regression coefficient of the ratio of weight gain (final weight ($FW$)—initial weight ($IW$)) to $FW$, to account for the effect of growth on gene expression, and ${\varepsilon }_{Fij}$ is a random residual. The trait phenotypes were scaled and centered (mean = 0 and SD = 1), such that the resulting regression slopes could be compared between traits. Genes with an expression that had significant regression coefficients on the trait at a false discovery rate (FDR) corrected *p*-value (*q*) < 0.05 were classified as trait-associated genes (TAG). TAG were analyzed for over-representation in KEGG pathways using the “kegga” function in the limma R-package [29]. Genes that encoded transcription factor proteins were identified by using the R-package SalMotifDB (https://salmobase.org/apps/SalMotifDB) [30], which interacts with a database of transcription factors for salmonids.

## Results and discussion

Efficient fish minimize the loss of deposited nutrients per unit growth, which is expected to affect the rate of change in the observed isotope profile. Using isotope data, individual phenotyping of feed efficiency is possible in Atlantic salmon even without obtaining registrations of individual feed intake. Estimates of heritability, genetic and phenotypic correlations among the studied traits and FCR were previously reported by Dvergedal et al. [4] and showed that the indicator feed efficiency traits IFCR/IFER in muscle had estimates of the genetic correlation with FCR on a tank level that were at the boundary of the parameter space (*r*_*g*_ ~ 1.0). However, ALC also showed a high genetic correlation with FCR on a tank level (*r*_*g*_ = − 0.90) but was less genetically correlated with growth-related traits than with IFCR/IFER on an individual level. Consequently, ALC may explain individual variation in feed efficiency that is not related to growth.

### Association analysis

To test whether phenotypes for feed efficiency such as ALC and IFCR/IFER variables are associated with SNPs, we performed a GWAS with a linear mixed-model algorithm, using indicator traits related to nitrogen and carbon metabolism, growth, and indicator traits for feed efficiency as phenotypes. To our knowledge, this is the first GWAS applied to indicator traits of feed efficiency and metabolism in muscle, liver, and adipose tissues of Atlantic salmon. Figure 1 illustrates significant associations between SNPs and traits of interest and significant associations with a −log10 (*p*) > 8 are in Table 1. The Manhattan plots for WG, RG, AMC, AMN, and ALN (Fig. 1a–f), showed two genomic regions that displayed a significant association on salmon chromosome (Ssa) 9, one between 13 and 31 Mb and one between 45 and 106 Mb.

**Table 1:** Single-nucleotide polymorphisms (SNP) with genome-wide significant (*p* < 10^–7^) associations with weight gain (WG), relative weight gain (RG), atom % ^13^C muscle (AMC), atom % ^15^N muscle (AMN), atom % ^13^C liver (ALC), and atom % ^15^N liver (ALN)

Trait	Chr	SNP	Base pair	A1	A2	Freq	b	se	*p*
WG	5	ctg7180001180119_4244_SCT	33597277	C	T	0.141	1.063	0.190	2.28E−08
	5	ctg7180001923117_1435_SAG	31819838	A	G	0.097	1.153	0.215	7.63E−08
	9	ctg7180001197157_4756_SAG	89517134	G	A	0.356	− 1.016	0.166	9.54E−10
	9	*ctg7180001818540_14626_SGT*	96172057	G	T	0.233	0.991	0.163	1.23E−09
	9	ctg7180001818540_11784_SCT	96174899	C	T	0.234	0.976	0.163	1.98E−09
	9	ctg7180001664612_2468_SCT	91842480	C	T	0.234	0.967	0.163	3.00E−09
	9	ctg7180001664612_1619_SAG	91841631	G	A	0.234	0.958	0.163	4.19E−09
	9	*ctg7180001545661_2981_SGT*	16416138	G	T	0.402	0.819	0.140	4.53E−09
	9	ctg7180001832507_11515_SCG	75470675	G	C	0.448	− 0.847	0.145	5.35E−09
	9	*ctg7180001868348_9058_SAG*	15945100	G	A	0.492	− 0.781	0.137	1.18E−08
	9	*ctg7180001628780_1051_SAG*	17214390	G	A	0.414	− 0.871	0.153	1.20E−08
	9	ctg7180001197157_4700_SAC	89517078	A	C	0.311	0.859	0.151	1.40E−08
	9	ctg7180001894494_11001_SAG	89556217	G	A	0.310	0.851	0.152	2.08E−08
	9	*ctg7180001911598_32299_SCT*	17106888	C	T	0.436	− 0.808	0.146	2.88E−08
	9	*ctg7180001802227_6890_SAC*	78211162	A	C	0.216	0.972	0.176	3.34E−08
	9	*ctg7180001806806_477_SAC*	78242149	A	C	0.216	0.969	0.176	3.67E−08
	9	*ctg7180001588841_1060_SGT*	86047894	G	T	0.234	0.855	0.155	3.77E−08
	9	*ctg7180001926947_6570_SGT*	81306559	T	G	0.247	0.885	0.161	3.86E−08
	9	ctg7180001380355_4100_SGT	96979010	G	T	0.422	0.721	0.133	5.50E−08
	9	ctg7180001921692_473_SGT	95413530	G	T	0.212	0.946	0.175	6.46E−08
	9	ctg7180001898405_11116	82286805	A	C	0.312	0.818	0.152	7.65E−08
	9	*ctg7180001678561_512_SGT*	90982168	T	G	0.335	0.769	0.143	7.99E−08
	9	*GCR_cBin45958_Ctg1_101*	19444985	G	A	0.386	0.825	0.154	8.16E−08
	9	ctg7180001859612_1950_SCT	106163425	T	C	0.426	0.837	0.157	9.45E−08
	20	ctg7180001900661_2996_SAG	29391087	A	G	0.472	0.651	0.116	1.97E−08
	20	ctg7180001900661_8312_SAC	29385772	A	C	0.472	0.632	0.116	4.75E−08
	20	ctg7180001403181_749_SGT	32398670	T	G	0.413	0.701	0.131	7.97E−08
RG	9	*ctg7180001820745_5080_SAG*	23240272	G	A	0.202	2.191	0.337	7.98E−11
	9	*ctg7180001604256_10823_SAG*	23113694	G	A	0.156	2.406	0.380	2.39E−10
	9	*GCR_cBin45958_Ctg1_101*	19444985	G	A	0.386	2.035	0.339	1.99E−09
	9	*ctg7180001789610_1630_SCT*	25039628	C	T	0.349	− 1.669	0.288	7.03E−09
	9	*ctg7180001841302_7054_SGT*	21739717	G	T	0.184	2.112	0.367	8.33E−09
	9	*ctg7180001841302_7076_SGT*	21739695	T	G	0.191	2.046	0.365	2.02E−08
	9	ctg7180001809374_3372_SCT	19466507	C	T	0.373	1.900	0.343	3.03E−08
	9	*ctg7180001847789_6042_SAG*	16428841	A	G	0.263	2.034	0.367	3.07E−08
	9	*ctg7180001857693_2711_SAG*	85086045	G	A	0.278	1.814	0.329	3.60E−08
	9	*ctg7180001545661_2981_SGT*	16416138	G	T	0.402	1.704	0.310	3.68E−08
	9	*ctg7180001468960_5703_SCT*	14756989	C	T	0.226	2.035	0.370	3.83E−08
	9	*ctg7180001931759_8478_SAG*	16329499	G	A	0.217	2.046	0.376	5.14E−08
	9	*ctg7180001898949_10269_SAG*	22638682	A	G	0.482	− 1.584	0.292	5.86E−08
AMC	9	*ctg7180001628780_1051_SAG*	17214390	G	A	0.415	− 0.013	0.002	1.04E−09
	9	*ctg7180001820745_5080_SAG*	23240272	G	A	0.202	0.013	0.002	2.45E−09
	9	*ctg7180001789610_1630_SCT*	25039628	C	T	0.349	− 0.011	0.002	5.22E−09
	9	*ctg7180001763729_3905_SAG*	15474718	A	G	0.283	0.013	0.002	8.27E−09
	9	*ctg7180001763729_4055_SGT*	15474568	T	G	0.283	0.013	0.002	1.14E−08
	9	*ctg7180001872184_4046_SAC*	59521565	A	C	0.267	− 0.012	0.002	1.69E−08
	9	*ctg7180001847789_6042_SAG*	16428841	A	G	0.261	0.013	0.002	1.91E−08
	9	*ctg7180001903467_551_SGT*	30327474	T	G	0.175	− 0.013	0.002	2.06E−08
	9	ctg7180001700380_482_SGT	15707203	G	T	0.282	0.013	0.002	2.08E−08
	9	*GCR_cBin45958_Ctg1_101*	19444985	G	A	0.385	0.012	0.002	3.47E−08
	9	*ctg7180001911598_32299_SCT*	17106888	C	T	0.436	− 0.011	0.002	3.78E−08
	9	ctg7180001903534_19011_SGT	13431163	T	G	0.283	− 0.013	0.002	3.98E−08
	9	*ctg7180001872184_453_SCT*	59517972	T	C	0.265	− 0.012	0.002	4.40E−08
	9	*ctg7180001604256_10823_SAG*	23113694	G	A	0.157	0.013	0.002	4.58E−08
	9	*ctg7180001343223_1775_SCT*	67759885	T	C	0.411	0.011	0.002	5.85E−08
	9	*ctg7180001545661_2981_SGT*	16416138	G	T	0.401	0.011	0.002	6.44E−08
	9	*ctg7180001833924_2266_SCT*	24557694	T	C	0.429	0.011	0.002	6.65E−08
	9	*ctg7180001794986_4059_SAC*	20044519	C	A	0.262	− 0.012	0.002	6.93E−08
	9	*ctg7180001898949_10269_SAG*	22638682	A	G	0.481	− 0.010	0.002	7.33E−08
AMN	9	*ctg7180001820745_5080_SAG*	23240272	G	A	0.202	0.034	0.005	3.24E−11
	9	*ctg7180001841302_7054_SGT*	21739717	G	T	0.184	0.036	0.006	1.47E−10
	9	*ctg7180001604256_10823_SAG*	23113694	G	A	0.157	0.037	0.006	1.87E−10
	9	*ctg7180001841302_7076_SGT*	21739695	T	G	0.192	0.033	0.006	1.33E−09
	9	*ctg7180001898949_10269_SAG*	22638682	A	G	0.481	− 0.027	0.004	1.51E−09
	9	*ctg7180001909530_3368_SAC*	30671958	A	C	0.289	0.032	0.006	1.69E−08
	9	*ctg7180001857693_2711_SAG*	85086045	G	A	0.278	0.028	0.005	2.38E−08
	9	*ctg7180001628780_1051_SAG*	17214390	G	A	0.415	− 0.028	0.005	2.78E−08
	9	*ctg7180001912930_10973_SAC*	59822403	C	A	0.446	− 0.026	0.005	4.30E−08
	9	*ctg7180001343223_1775_SCT*	67759885	T	C	0.411	0.026	0.005	4.62E−08
	9	*ctg7180001911598_32299_SCT*	17106888	C	T	0.436	− 0.026	0.005	8.19E−08
	9	*ctg7180001254975_135_SCT*	30005989	T	C	0.328	0.017	0.003	9.72E−08
ALC	12	ctg7180001233434_1518_SCT	45935004	C	T	0.418	− 0.009	0.001	1.79E−10
	12	ctg7180001878331_16006_SAG	67415693	A	G	0.411	− 0.008	0.001	3.95E−10
	12	ctg7180001589944_2780_SAC	67420787	C	A	0.410	− 0.008	0.001	5.82E−10
	12	ctg7180001917752_6118_SAG	68229784	A	G	0.342	− 0.008	0.001	6.01E−09
	12	ctg7180001926810_6994_SGT	59975663	T	G	0.445	− 0.008	0.001	8.58E−09
	12	ctg7180001924417_6623_SAC	66355729	A	C	0.178	− 0.010	0.002	1.38E−08
	12	ctg7180001863800_134_SAC	54013376	C	A	0.495	− 0.008	0.001	1.71E−08
	12	ctg7180001930970_12364_SCT	34715679	C	T	0.460	0.007	0.001	1.86E−08
	12	ctg7180001787629_3714_SGT	63703424	G	T	0.441	− 0.008	0.001	1.92E−08
	12	ctg7180001926810_5584_SCT	59974253	C	T	0.435	− 0.008	0.001	2.17E−08
	12	ctg7180001759831_1827_SCT	45916216	C	T	0.364	− 0.007	0.001	2.33E−08
	12	ctg7180001481690_187_SAG	73548289	G	A	0.438	− 0.008	0.001	3.22E−08
	12	ctg7180001912956_2486_SGT	36748230	T	G	0.416	− 0.007	0.001	3.25E−08
	12	ctg7180001926810_6801_SAG	59975470	A	G	0.444	− 0.007	0.001	3.28E−08
	12	ctg7180001899463_4736_SCT	45925520	C	T	0.365	− 0.007	0.001	3.78E−08
	12	ctg7180001874153_6984_SAC	59968424	C	A	0.444	− 0.007	0.001	3.82E−08
	12	ctg7180001903261_15275_SCT	36741726	T	C	0.419	− 0.007	0.001	4.80E−08
	12	ctg7180001802518_8127_SAG	38630722	G	A	0.264	− 0.008	0.002	5.60E−08
	12	ctg7180001895532_9980_SAC	52536172	C	A	0.150	− 0.010	0.002	8.47E−08
ALN	9	*ctg7180001820745_5080_SAG*	23240272	G	A	0.202	0.034	0.005	1.71E−10
	9	*ctg7180001604256_10823_SAG*	23113694	G	A	0.157	0.037	0.006	3.95E−10
	9	*ctg7180001902776_3165*	44544043	A	G	0.030	− 0.082	0.013	1.00E−09
	9	ctg7180001297112_1053_SAC	44743511	C	A	0.029	− 0.079	0.013	3.51E−09
	9	ctg7180001846444_1581_SAG	27624708	G	A	0.050	− 0.060	0.010	3.64E−09
	9	*ctg7180001898949_10269_SAG*	22638682	A	G	0.481	− 0.027	0.005	6.84E−09
	9	ctg7180001841823_6182_SGT	27830435	G	T	0.050	− 0.059	0.010	8.19E−09
	9	ctg7180001841823_8622_SAG	27832875	A	G	0.051	− 0.058	0.010	9.34E−09
	9	ctg7180001897675_6237_SCG	26358698	C	G	0.050	− 0.058	0.010	1.04E−08
	9	*ctg7180001841302_7076_SGT*	21739695	T	G	0.192	0.033	0.006	1.28E−08
	9	*ctg7180001841302_7054_SGT*	21739717	G	T	0.184	0.033	0.006	1.39E−08
	9	ctg7180001516979_6848_SCT	26634813	T	C	0.050	− 0.058	0.010	1.54E−08
	9	ctg7180001905112_13597_SAC	25462494	C	A	0.061	− 0.052	0.009	1.57E−08
	9	ctg7180001516979_7200_SCT	26635165	C	T	0.050	− 0.057	0.010	2.04E−08
	9	GCR_cBin3500_Ctg1_117	27426856	C	G	0.081	− 0.044	0.008	3.60E−08
	9	*ctg7180001927229_6536*	84462126	A	G	0.448	0.027	0.005	4.58E−08
	9	ctg7180001905111_1804_SAC	25477875	C	A	0.062	− 0.050	0.009	4.77E−08
	9	ctg7180001322796_3617_SGT	50798901	G	T	0.113	− 0.037	0.007	6.77E−08
	9	ctg7180001796082_675_SAC	19872408	C	A	0.148	− 0.035	0.006	7.31E−08

SNPs are ranked within trait and chromosome. Significant associations that are common between traits are indicated in italic characters

A1: reference allele; A2: alternative allele; Freq: frequency of A1; b: SNP effect (the effect of increasing the genotype with one extra reference allele) with associated standard error (se) and p-value (p)

Many of the significant SNPs were shared between traits (Table 1). On Ssa9, four to 10 significant SNPs were in common between the traits WG, RG, AMC, AMN, and ALN, which may be because all these traits are closely associated with growth. Also, two SNPs on Ssa3, four on Ssa5, and 12 on Ssa20 were associated with WG, all with a −log10 (*p*) > 6.03 (Fig. 1a), while one SNP on Ssa11, one Ssa12, and one Ssa15 associated with AMN (Fig. 1d), and one SNP on Ssa20 with ALN (Fig. 1f). No significant SNP associations with AAC were found at the evaluated age (~ 10 months), which might be because lipid deposition is at its maximum beyond this age, during the grow-out phase in the sea (~ 1.5–4 kg). Therefore, we cannot rule out significant SNP associations with lipid deposition at a later life-stage in salmonids [31–33].

Genome-wide significant SNPs that were associated with growth-related traits, such as WG, RG, AMC, AMN, and ALN, were mainly located on Ssa09. Gutierrez et al. [34], who mapped QTL for body weight in Atlantic salmon at different stages of life, reported genome-wide significant SNPs (QTL) on Ssa09 for individuals of the same age as in this study (~ 10 months). They also detected chromosome-wide significant SNPs on Ssa20, but did not find any common significant SNPs at different stages of life. Baranski et al. [35] argued that the large number of QTL for growth-related traits that act at different stages of life implies that bodyweight is a polygenic trait in Atlantic salmon. However, as growth of salmons occurs mostly during the saltwater phase, the commercial interest of a QTL for body weight in the freshwater-phase is most likely limited.

Nineteen genome-wide SNPs (−log10 (*p*) > 8) were also found on a Ssa12 region that affects ALC (Fig. 1e). Dvergedal et al. [4] showed that ALC had low genetic correlations with growth (WG and RG) (*r*_*g*_ = 0.16 and 0.12, respectively, with standard errors of 0.12 and 0.14), which suggest that ALC might be genetically independent of growth (WG and RG). From an economic point of view, a QTL that is involved in improving feed efficiency without being associated with growth would be highly relevant and would add valuable information that cannot be captured by recording the growth of the individuals only. However, the SNPs of interest were spread over a 40-Mb region (between 34 and 73 Mb) that contains 770 genes (NCBI search).

Only three genome-wide significant SNP associations were found for the indicator trait IFER_AMN, on Ssa06, Ssa23, and Ssa27 (Fig. 2d), and none for IFCR_AMC, IFCR_AMN, and IFER_AMC (Fig. 2a–c), which suggests that all IFCR and IFER variables are polygenic traits. In fact, Dvergedal et al. [4] reported low heritability estimates for these indicator traits of feed efficiency, but this study included few families, and thus if a putative QTL does exist in the population it might not be represented in the 23 families analyzed. This means that a larger number of families should be studied, which might increase the number of haplotypes represented in the data and increase the possibility to accurately pinpoint the position of a QTL. Moreover, with strong family structures, long stretches of the same haplotype that are identical-by-descent [21] are likely to occur, which can result in the wide peaks, and in reducing the probability of finding significant SNPs for the indicator traits IFCR/IFER in these data.

With few but large families, our dataset is appropriate to detect QTL but not to fine map them, since longer DNA segments surrounding the QTL may be significant. For the traits for which significant QTL were detected, the shape of the QQ-plots (Fig. 3a–f) can be explained by the substantial linkage disequilibrium (LD) in the population as a result of the limited effective population size. For AAC (Fig. 3g) and the IFCR/IFER traits (Fig. 4a–d) for which no significant QTL were found, the shape of the QQ plots was as expected.

## RNAseq analysis

**Fig. 5:**
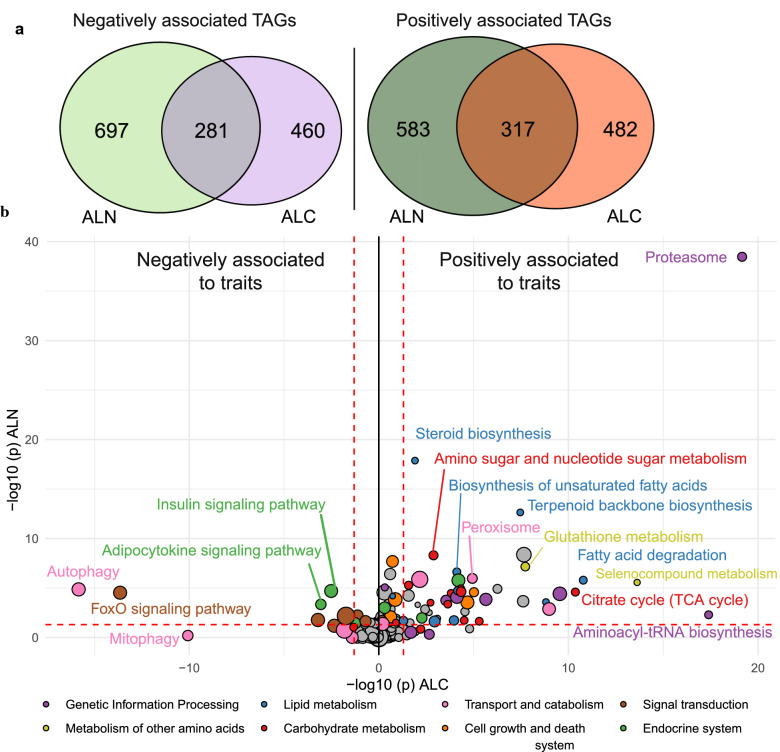
**a** Number of genes the expression of which was associated with atom % ^13^C liver (ALC) and atom % ^15^N liver (ALN) and **b** KEGG pathways enriched among positively (right) and negatively (left) associated genes for ALC (x-axis) and ALN (y-axis). Size indicates the number of genes in the KEGG pathway. The *p*-value cutoffs of 0.05 are indicated as red dashed lines

To assess the effects of ALC and ALN on gene expression, we sequenced RNA from the liver of 184 fish, which represented a subset of all the families included in this experiment. Regression analysis using ALC as a covariate identified 799 TAG with positive associations of gene expression and ALC and 741 TAG with negative associations (FDR corrected *p*-value (*q*) < 0.05). For ALN, we identified 900 TAG with positive associations and 978 TAG with negative associations (*q* < 0.05). Three hundred and seventeen genes were shared among the TAG with positive associations between ALC and ALN and 281 TAG were shared among the TAG with negative associations (Fig. 5a).

KEGG enrichment analysis of the positive and negatively associated TAG revealed more enriched KEGG pathways (*p* < 0.05) among the positively associated TAG (59 for ALC and 88 for ALN) than among the negatively associated TAG (24 for ALC and 35 for ALN). All TAG and enriched KEGG pathways are in Additional file 1: Table S1 and Additional file 2: Table S2 respectively. “Proteasome” was the most enriched pathway for both ALC and ALN. Since the stable nitrogen and carbon isotopes ingested by the fish were mainly in the form of protein, higher proteasome levels likely accelerate the breakdown and incorporation of stable isotopes in tissues of the fish. Another highly enriched pathway for both traits was the “TCA cycle”, which is central to carbon metabolism and converts carbon from proteins, lipids, and carbohydrates into acetyl-CoA for fatty acid and cholesterol biosynthesis and NADPH for a variety of cellular processes (Fig. 5b). Two amino acid metabolism pathways “Glutathione metabolism” and “Selenocompound metabolism” were positively associated with high ALC and ALN. Both glutathione and selenocysteine are key components of glutathione peroxidases, which represent a family of antioxidant enzymes involved in counteracting the negative effects of reactive oxygen species produced during oxidative phosphorylation [36]. In addition, we observed that the cholesterol biosynthesis pathways “Terpenoid backbone synthesis”, “Steroid biosynthesis” and the fatty acid metabolism pathways “Fatty acid degradation” and “Fatty acid biosynthesis” were positively associated with ALC and ALN (Fig. 5b). Cholesterol and fatty acids are key components of cell membranes, and a higher activity of these pathways could contribute to a higher level of ^13^C incorporation in tissues. The pathways that were negatively associated with ALC and ALN, included several signaling pathways and also the “Autophagy” and “Mitophagy” pathways (Fig. 5b). Taken together, our results suggest that individuals with high rates of liver carbon and nitrogen metabolism (ALC and ALN) convert protein from the feed into body tissue more efficiently, primarily because of higher proteasome gene expression, but also because of elevated oxidative phosphorylation and lipid metabolism.

To identify potential genes that drive differences in gene expression that are associated with ALC and ALN, we cross-referenced our list of TAG within the quite large regions that were identified on Ssa9 and 12 and that harbored 2532 genes. Among these genes, 177 were in our list of TAG, of which seven are known transcription factors (TF) (Table 2). Four and three of these TF had significantly (*p* < 0.05) negative associations with ALC and ALN, respectively, which agrees with our KEGG analysis that showed that negatively associated pathways were the most enriched in signaling pathways (Fig. 5b). Two of the TF belong to the FoxO family, which is involved in a range of physiological processes, including insulin signaling, autophagy, and proteasome regulation [37].

**Table 2:** Transcription factors that were enriched in the QTL regions on chromosomes (Chr) 9 and 12 with their start and end position on the chromosome

Transcription factor	Gene ID	Chr	Start	End	ALC^a^		ALN^b^	
					b_1_	*q*	b_1_	*q*
Steroid hormone receptor ERR2-like	LOC106611051	9	22198787	22262832	− 0.08	0.45	− 0.26	0.02
Hepatocyte nuclear factor 3-alpha-like	LOC106611236	9	34548245	34551243	− 0.09	0.03	− 0.04	0.65
Uncharacterized LOC106611247	LOC106611247	9	35172348	35208430	− 0.05	0.34	− 0.12	0.03
Forkhead box protein N3-like	LOC106611366	9	42014771	42135841	− 0.07	0.57	− 0.27	0.03
Gastrula zinc finger protein XlCGF26.1-like	LOC106612579	9	106204316	106211994	− 0.13	0.02	− 0.09	0.29
Transcription factor SOX-4-like	LOC106565395	12	52077963	52084651	− 0.26	0.04	− 0.33	0.06
Interferon regulatory factor 6-like	LOC106565674	12	59247924	59255854	− 0.17	0.00	− 0.14	0.06

^a^Atom % ^13^C liver (ALC)

^b^Atom % ^15^N liver (ALN)

b_1_ is the fixed regression coefficient of the phenotype for the trait (ALC or ALN) and *q* is the false discovery rate (Benjamini–Hochberg corrected)

## Conclusions

In this study, we identified two important QTL for performance of Atlantic salmon in freshwater, one for growth on Ssa9, and one for carbon metabolism in the liver on Ssa12. Carbon metabolism in the liver was closely related to FCR at the tank level. However, we were not able to accurately map the putative QTL. For the IFCR/IFER phenotypes that were derived from the ratios between the fraction of stable isotopes (^15^N and ^13^C) in muscle and growth, no convincing QTL were detected. Transcriptomic analysis revealed a positive association of ALC and ALN with the ability to convert protein from the feed into body tissue more efficiently, primarily through the increased expression of proteasome, lipid, and carbon metabolic pathways in the liver. In addition, we identified seven transcription factors that were associated with ALC or ALN and that were located in the QTL identified.

## Supplementary information

**Additional file 1: Table S1.** An overview of all trait-associated genes (TAG) with atom % ^13^C (ALC) and atom % ^15^N (ALN) in the liver.

**Additional file 2: Table S2.** An overview of all enriched KEGG pathways associated with atom % ^13^C (ALC) and atom % ^15^N (ALN) in the liver.

## Data Availability

The genotypic data are owned by AquaGen AS and are used under license for this study, and thus are not publicly available. Phenotypic data can be made available on request.
